# Complete Genome Sequence of *Pragia fontium* 24613, an Environmental Bacterium from the Family *Enterobacteriaceae*

**DOI:** 10.1128/genomeA.00740-15

**Published:** 2015-07-09

**Authors:** Kateřina Snopková, Karel Sedlář, Juraj Bosák, Eva Chaloupková, Ivo Provazník, David Šmajs

**Affiliations:** a Department of Biology, Masaryk University, Brno, Czech Republic; b Department of Biomedical Engineering, Brno University of Technology, Brno, Czech Republic

## Abstract

The complete genome sequence of *Pragia fontium* 24613 was determined using PacBio RSII, Roche 454, and SOLiD sequencing. A total of 3,579 genes were predicted, including 3,338 protein-coding sequences and 146 pseudogenes. This is the first whole-genome sequence of a strain belonging to the environmental genera of the family *Enterobacteriaceae*.

## GENOME ANNOUNCEMENT

*Pragia fontium* is a Gram-negative, mesophilic, rod-shaped, motile bacterium from the family *Enterobacteriaceae*. While one isolate originated from a stool sample from a healthy woman, all other isolates came from water wells or water pipes. The genus *Pragia* contains only one species, *P. fontium*, which was first described in Czechoslovakia in 1988 (1). Strain *P. fontium* 24613 was isolated from a water pipe in 1983 (1). *P. fontium* produces H_2_S and oxidizes gluconate, which distinguishes this species from other enterobacterial H_2_S producers. The genus *Pragia* is one of the few genera from the family *Enterobacteriaceae* that is isolated almost exclusively from environmental samples.

The total DNA genome of *P. fontium* 24613 was sequenced using PacBio RSII (GATC Biotech, Inc., Constance, Germany), Roche 454 (Roche Genome Sequencer FLX; Eurofins Genomics, Inc., Ebersburg, Germany), and SOLiD V3Plus (SeqOmics, Inc., Mórahalom, Hungary) platforms. PacBio single-molecule real-time (SMRT) Analysis version 2.3 was used for PacBio raw read treatment (covering ≈300 Mbp). HGAP software (2) was used for *de novo* genome assembly, with ≈30× coverage of self-corrected reads with length >4,746 bp. Contig accuracy was enhanced with the Quiver tool using the entire read set (70× coverage). The contigs were ordered according to an optical map (OpGen, Inc., Gaithersburg, MD, USA), which compared the AflII restriction pattern of the *P. fontium* chromosome to the *in silico*-obtained contigs. Contig overlaps were manually trimmed using the Geneious software (3), and the remaining gaps were filled with Sanger sequencing of the PCR products. Roche 454 reads (11× coverage) and SOLiD reads (50× coverage) were used for increasing accuracy and final corrections of the whole-genome sequence. Ori-Finder (4) was used for *oriC* detection, and the genome sequence was numbered from *oriC*. Annotation was performed with the NCBI Prokaryotic Genome Annotation Pipeline (http://www.ncbi.nlm.nih.gov/genome/annotation_prok/). Open reading frames were predicted using GeneMarkS+ and ProSplign. Ribosomal RNAs, tRNAs, and small noncoding RNAs (ncRNAs) were identified using BLASTn, tRNAscan-SE, and Cmsearch.

*P. fontium* 24613 was found to contain only chromosomal DNA; no plasmids were detected. The whole-genome sequence of *P. fontium* 24613 comprised 4,094,629 bases, and the G+C content was 45.4%. In total, 3,579 genes were predicted, including 3,338 protein-coding sequences (CDSs) and 146 pseudogenes. Twenty-two rRNA and 72 tRNA genes were also identiﬁed in the genome sequence. The genome size was only slightly smaller than the genome sizes of closely related strains of the genera *Yersinia* and *Pectobacterium*; the G+C content was quite similar as well. Several genes predicted in the *Pragia* genome are similar to genes detected in plant pathogens and growth-promoting rhizobacteria (5). The genome of the *Pragia* strain will be analyzed in detail and compared to other *Enterobacteriaceae* genomes.

### Nucleotide sequence accession number. 

The *P. fontium* 24613 whole-genome sequence has been deposited in GenBank under the accession no. CP010423.
